# Occurrence of mosaic trisomy 22 and pericentric inversion of chromosome 9 in a patient with a good prognosis

**DOI:** 10.1186/s12920-023-01709-2

**Published:** 2023-11-13

**Authors:** Aline Nardelli, Larissa Valéria Laskoski, Andressa Fernanda Luiz, Maruhen Amir Datsch Silveira, Luciana Paula Grégio d’Arce

**Affiliations:** 1 Hospital Universitário do Oeste do Paraná, Western Parana State University, Avenida Tancredo Neves 3224, Santo Onofre, Cascavel, Paraná Brazil; 2 Centre de Recherche sur le Cancer de l’Université Laval, 9 Rue McMahon, Québec, QC G1R 3S3 Canadá

**Keywords:** Chromosome abnormalities, Genetic diagnosis, Mosaicism, Chromosome 22 trisomy

## Abstract

Complete trisomy 22 is a rare chromosomal condition that is incompatible with life. However, mosaic trisomy 22 usually has prolonged survival compatibility and may present a good prognosis depending on the tissues affected. Herein, we described a male patient with the occurrence of mosaic trisomy 22 associated with the inversion of chromosome 9, with karyotype 47, XY, inv (9) (p11q13), + 22 [5] / 46, XY, inv(9) (p11q13) [45] and arr 22q11.1 ~ q13.33(16,417008-51,219,009)x2 ~ 3. It is not possible to infer, in general, the clinical characteristics associated with mosaic trisomy 22. However, the patient presented common clinical features observed in reported cases (in parentheses the percentage observed comparing all reported cases): facial dysmorphia (100%), delay in motor development/growth (82%), cardiac abnormalities (73%), ear abnormalities (55%) and facial and/or body asymmetry (55%), in addition to hypotonia, skin spots, hypoplastic nails. Given the survival and quality of life associated with multidisciplinary treatment, it can be concluded that the patient has a good prognosis. Conclusively, we’re presenting the occurrence of mosaic trisomy 22 and chromosome 9 inversion in the patient with favorable prognosis. Thus, this study proposed a guide which should be inserted in databases of rare genetic conditions to help genetic counselors define mosaic trisomy 22 diagnosis.

## Introduction

Chromosome 22 trisomy is a rare chromosomal condition observed in 2–5% of spontaneous abortions [1, 2]. Recently, trisomy 22 was observed in only 4 of 330 miscarriage samples, with no case of mosaic trisomy 22 [3]. Complete trisomy 22 is characterized by an extra third copy (where normally there should be only two copies) of the chromosome 22 in every cell in the body [4, 5]. On the other hand, mosaicism refers to the occurrence of two or more genomes derived from a single zygote, which may be germinal or postzygotic [6]. Therefore, the term “mosaic” indicates that some cells contain the extra chromosome 22, while others have the normal chromosome pair, with different levels of mosaicism between the tissues [7]. Phenotypically, while most individuals affected with complete trisomy 22 die before, or shortly after, birth, due to severe birth defects [4, 5], mosaic trisomy 22 individuals show prolonged survival compatibility, confirming the importance for genetic counseling of recognizing full trisomy 22 from the mosaic form [8].

Mosaic trisomy 22 was first described by Schinzel in 1981 [9], and about 20 live-born children with this condition are currently reported. Back then, authors suggested prevalent clinical presentations, as webbed neck, abnormal ears, cardiac disorders, microcephaly, and developmental delay [9]. Nowadays, variable clinical features were described, including growth restriction, facial anomalies, congenital anomalies, limb malformations, dysmorphic features, and hemihyperplasia. Neurodevelopmental outcome ranges from normal to severe intellectual delay [10, 11].

Herein, we report a current 11-year-old male patient with mosaic trisomy 22 and pericentric inversion of chromosome 9. This study documents the physical characteristics, health history, gestational data, and information about the treatment received by the patient at the Center for Craniofacial Anomalies Care and Research, at University Hospital Western Parana, located in the city of Cascavel, Parana, Brazil.

## Case report

Male patient, current 11 years old, born at term, 39 weeks and 5 days old, cesarean section, weight 2.810 kg (< 10th percentile), height 44 cm (< 3rd percentile), head circumference 35 cm (25th-10th percentile) and Apgar score 8/9. Second child of a non-consanguineous marriage, mother was 23 years old and father 26 years old when he was born, both disease-free. It was described that the pregnancy occurred with some complications, including tiredness, and tingling in the upper limbs, use of uterine relaxant (isoxsuprine hydrochloride) until the 3rd month of pregnancy due to uterine contractions and loss of amniotic fluid around the 8th gestational month.

Mother reported using folic acid and ferrous sulfate from the 7th week onwards; both parents did not use alcohol, cigarettes, or other teratogenic agents during pregnancy.

Although the mother had strict prenatal care checkups, no health professional referred her for amniocentesis or any type of genetic testing. This is possibly due to the standard guidelines used in the Brazilian public health system, where NIPT is usually not covered. Therefore, no genetic test was performed before birth.

### Birth conditions

Regarding the clinical findings, the patient has prominent ears, low set ear with posterior rotation, prominent forehead, high myopia, ptosis, epicanthus, strabismus, downward slanting palpebral fissure retrognathia, freckles on the face, curve and webbed neck, asymmetry in the left hemi body, hypotony, kyphosis and scoliosis, café au lait spots on the back and upper limbs, hypoplastic toenails, bilateral cryptorchidism (surgically corrected), delayed growth, congenital heart defects and hypothyroidism.

Surgeries performed on the patient after birth: correction of interatrial communication at 11 months old, orchidopexy at 5 years old, correction of strabismus, oculomotor nerve and ptosis in the left eye, tonsillectomy at 10 years old.

**Fig. 1:**
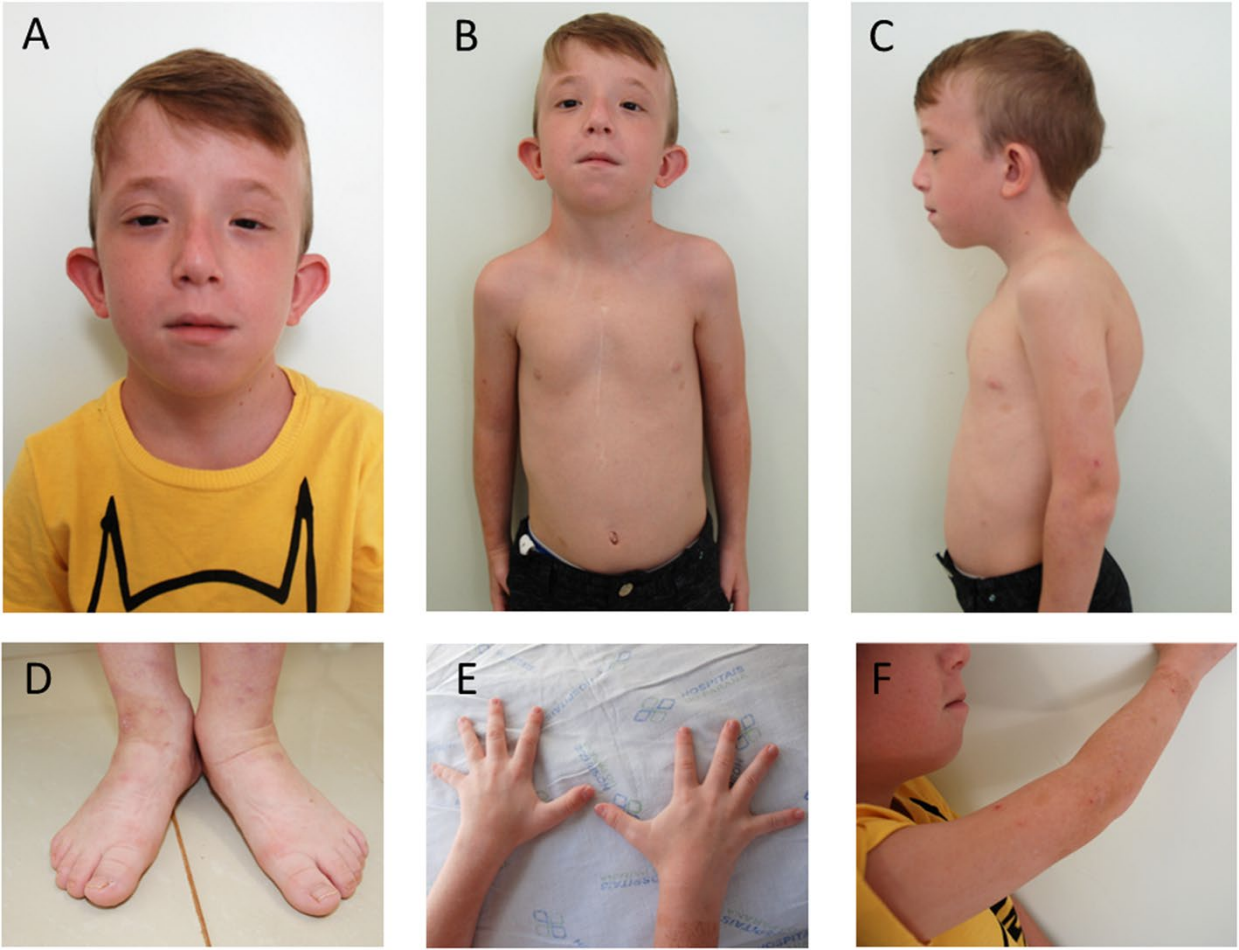
Proband images: **A** facial: left eye ptosis, micrognathia, long philtrum, protruding ears, low set ear; **B** ventral position of the trunk; **C** lateral trunk: low set ear, kyphosis; **D** feet: hemi body asymmetry, café au lait spots; **E **fingers of the hands; **F** upper limb: café au lait spots

After radiographs, a bone age of 6 years was detected when the patient was 9 years old. Patient makes use of growth hormone and hypothyroidism medication, has lactose intolerance, allergy to insect stings and asthmatic bronchitis. The proband phenotype is shown at 9 years of age (Fig. 1).

### Patient’s developmental history

Since birth, the patient always showed growth below the lowest percentile of the curve. At the age of 11, the patient was 1.21 m tall (< P0.1 – very low height for age - WHO) and weighed 22 Kg (< P0.1 - very low weight for age - WHO). At 6 months of age, he showed neuropsychomotor development delay and started physical therapy. He walked with 3 years old and spoke at 3 years and 6 months. He remained in therapeutic follow-up with fortnightly appointments including motor physiotherapy and postural kinesiotherapy. Despite hypotonia and movement restriction due to the lack of fine motor coordination, the patient is active, playing soccer and practicing swimming. He became literate at 7 years old and has good school performance. As attested by a multidisciplinary team, the patient has the expected intellectual and adaptive function for his age, without difficulties in concentration, attention, language, or visual processing of information. He does not have discrepancies between potential and school performance that led to further referral to specialists.

### Cytogenetic analysis

As previously explained, the mother underwent prenatal care following a high-risk pregnancy protocol. However, the local network does not include NIPT tests [12], meaning the proband was referred for genetic testing only after birth. Herein, karyotyping is the first-tier test available in our center as we follow guidelines from the Unified Health System (SUS). SUS is maintained by the federal government, which set all the health actions and services provided by public institutions.

Peripheral blood was collected from both parents and proband. Karyotype (GTG-banding) of peripheral blood lymphocytes was performed according to ISCN 2020 [13]. Chromosomal studies of the parents showed karyotypes 46, XX and 46, XY, inv(9) (p12q13) respectively for the mother and father. The pericentric inversion of the heterochromatic region was visualized in one of the homologs of chromosome 9 in all analyzed cells from the father.

Cytogenetic analysis of the patient revealed a karyotype, 47, XY, inv(9) (p11q13), + 22 [5] / 46,XY, inv(9) (p11q13) [45]. The presence of two cell lines was revealed: the first had three chromosomes 22 in 5 of the 50 cells analyzed, and the second lineage showed a normal karyotype in 45 of the 50 cells analyzed. Both strains showed a pericentric inversion of the heterochromatic region on one of the homologs of chromosome 9. This inversion is a variant inherited from its father, found in the general population with no known clinical significance on carriers [14]. As karyotyping suggested mosaicism, the proband’s family opted for further investigation using the CGH-array. CGH-array (750k) was performed using peripheral blood to search for microdeletions/microduplications. The test result was arr 22q11.1 ~ q13.33(16,417008-51,219,009)x2 ~ 3 confirms the mosaic involving the entire long arm of chromosome 22.

After the CGH-Array results and further genetic counseling, the family opted not to proceed with any further investigation, and no further testing in different tissues was performed, mostly due to the high cost of these tests in Brazil and the current good prognosis of the patient. However, the team agreed the data was sufficient and of high quality for the mosaicism diagnosis involving chromosome 22.

## Discussion

Here, the 11-year-old boy described in this study presents two different chromosomal abnormalities: de novo mosaic trisomy 22 in 10% of the analyzed cells, and pericentric inversion of chromosome 9 inherited from the father, present in all cells. Studies show that chromosomal abnormalities can be diagnosed before or after birth. NIPT can be used as a screening method in all pregnancies, but an invasive confirmatory test through amniocentesis or chorionic villus sampling is strongly recommended in specific cases [15]. Nonetheless, the family pursued for genetic counseling only at age 3 and the diagnosis was obtained by karyotyping, followed by CGH-array.

The best-known structural anomaly in chromosome 22 is the 22q11.2 deletion [16] and the occurrence of mosaic trisomy 22 and chromosome 9 inversion in a long-living patient is a rare case not previously documented. As far as we know, only one case combining total trisomy 22 and inv(9) has been described, a girl with karyotype 47, XX, inv(9) (p11q13), + 22, who died 3 days after birth, with intrauterine growth restriction, microcephaly, wide nose and flat bridge and hypertelorism [17], an event that is notably incompatible with life.

Alternatively, there are isolated information from patients with chromosome 9 inversion or chromosome 22 trisomy, with different clinical conditions. Chromosome 9 inversion is considered one of the most common chromosomal anomalies, observed in 1–3% of the general population [18]. Despite the inversion of chromosome 9 being identified as a normal variant, many studies show conflicting results regarding the chromosomal association between inv(9) and abnormal clinical conditions [15, 19], including the effects on fertility [20, 21], on female reproductive capacity [22] and spontaneous abortions [23]. Collectively, these studies suggest that pericentric inversion on chromosome 9 might be associated with fertility problems.

However, the negative impact on the individual is not limited to reproductive capacity. In a study to evaluate the clinical impact of the pericentric inversion of chromosome 9 (p11q13) in patients, several congenital anomalies were observed, but were not significantly different from the general population, which, according to the author, does not denote a pathogenic mutation [24]. On the other hand, a recent study concluded that pericentric inversion of chromosome 9 is associated with congenital anomalies, growth retardation, infertility, recurrent miscarriages, and cancer [19]. Collectively, these data suggest the clinical characteristics observed in patients may be related to the breakpoint during inversion, since chromosome 9 has the highest level of structural variability, needing further studies on the regions involved to understand the phenotypic effects and its consequences.

Herein, the chromosomal inversion event of chromosomes 9 observed in the patient under study corresponds to the p12q13 region inherited from the father, that had no associated phenotypic effects. Although inv(9) is considered a normal variant of the human karyotype, Amiel et al. (2001) [25] suggests that when there is an inv(9) it can lead to non-disjunction during meiosis. Indeed, it is observed a higher incidence of children with Down syndrome in parents with inv(9) [26]. The patient under study has mosaicism of chromosomes 22, a temporally later event, that may be associated with inv(9). This data is reinforced by an in vitro fertilization study, which verified a lower cleavage rate was observed in patients with inv(9), which may lead to interchromosomal effects with a higher incidence of mitotic disorders, probably associated with aneuploidies [27]. Finally, it has been reported that co-existence of pathogenic gene variants with apparently harmless variants may influence the phenotypic outcome of the disease [28].

Complete trisomy 22 represents 18.5% of chromosomal abnormalities resulting in spontaneous abortions [29, 30]. In some countries, when trisomy 22 is confirmed in the prenatal period, termination of pregnancy could be considered [31]. Therefore, it is to suggest that the variability of clinical signs of the disease reflects the proportion of cells with trisomy in different tissues. Additional information from studies revealed 47,XX,+22[5]/46,XX[25] in cardiomyocytes and 47,XX,+22[6]/46,XX[44] in cutaneous fibroblasts from one patient [10], and 47,XY,+22[28]/46,XY[72] in lymphocytes, and 47,XY + 22 in fibroblasts from another patient [32]. In the last study, it is possible to notice the increased severity of malformations (probably associated to the complete trisomy 22 observed in fibroblasts), including atrial and ventricular septal defect, right ventricle was reported small and patent ductus arteriosus, a right-to-left shunt and pulmonary hypertension. Indeed, due to the severity of the congestive heart failure, surgery was performed to ligate the ductus arteriosus and band the pulmonary artery, but the child died of cardiopulmonary failure [32]. Finally, a patient 47,XX,t(4;6)(q33;q23.3),+ 22 was described, which died at 35 days of age from complex heart disease and renal failure [4]. The proband manifested intrauterine growth retardation (IUGR), single umbilical artery, cranial abnormalities, short neck, cleft lip and palate, dysmorphic ears, hypoplastic nipples, digital malformation, congenital heart defects, dysplastic kidneys, and genital anomalies. Altogether, the events observed in complete trisomy 22 are also observed in mosaic trisomy 22, with decreased severity depending on the tissue affected.

Herein, the patient under study presented common characteristics already described in patients with mosaic trisomy 22 (Table 1), including facial dysmorphia (observed in 100% of the described cases), delay in motor development/growth (82%), cardiac abnormalities (73%), ear abnormalities (55%) and facial asymmetry and/or or body (55%). Other common features are intrauterine growth retardation, variable hypotonia, hypoplastic/dysplastic skin and nail patches, epicanthus, hypertelorism, and poor ear implantation (45%). On the other hand, it is possible to observe many anomalies and dysmorphisms exclusively described for few patients within the papers. Allergy to insects, asthma, bronchitis and lactose intolerance were exclusively described in our study. Due the rarity of this genetic condition, it is no surprise the few numbers of cases reported. Nonetheless, it is possible to observe a trend in the clinical characteristics, which suggest a clinical profile to be inserted in the databases of rare genetic conditions, given the need for the availability of information to aid health professionals.

**Table 1:** Characteristics commonly found in patients with mosaic trisomy of chromosome 22

Features	1		2	3	4	5	6	7	8	9	10	11	Total number of patients	Feature frequency/ total patients(%)
Feature frequency/ patient(%)		50	42	30	26	26	24	22	22	26	18	18		
Facial dysmorphia		x	x	x	x	x	x	x	x	x	x	x	11	100
Developmental delay/ growth retardation		x	x		x	x	x	x	x	x		x	9	82
Cardiac abnormalities		x	x	x	x		x	x			x	x	8	73
Flat nasal bridge			x	x	x	x		x	x	x			7	64
Ear abnormalities			x	x		x	x		x			x	6	55
Body/facial asymmetry		x	x		x		x			x		x	6	55
Intrauterine growth retardation							x	x	x	x	x		5	45
Epicanthus		x		x					x		x	x	5	45
Hypertelorism			x	x		x	x				x		5	45
Hypotonia (variable)		x			x			x	x		x		5	45
Low set ear		x	x	x				x		x			5	45
Spots on the skin		x	x			x			x	x			5	45
Dysplastic/hypoplastic nails		x	x		x	x					x		5	45
Genital abnormalities		x		x	x			x					4	36
Ears with posterior rotation		x	x	x		x							4	36
Body/member/face hypoplasia		x	x	x									3	27
Cleft palate				x	x		x						3	27
Prominent forehead		x		x					x				3	27
Microcephaly						x			x		x		3	27
Micrognathia/retrognathia		x		x			x						3	27
Webbed neck		x	x	x									3	27
Ptosis		x						x		x			3	27
Congenital brain abnormalities				x							x		2	18
Anterior ectopic anus								x				x	2	18
Scoliosis/kyphosis		x					x						2	18
Clinodactyly			x									x	2	18
Intellectual disability						x				x			2	18
Down-slanting palpebral fissures		x								x			2	18
Squint		x								x			2	18
Hearing loss					x					x			2	18
Hydronephrosis					x					x			2	18
Joint hypermobility						x	x						2	18
Hypothyroidism		x			x								2	18
Thin lips			x			x							2	18
Myopia		x							x				2	18
Anteverted nares			x					x					2	18
Insect sting allergy		x											1	9
Asthma		x											1	9
Downward turning of the mouth												x	1	9
Bronchitis		x											1	9
Tapering fingers			x										1	9
Long soft philtrum			x										1	9
Hemangioma			x										1	9
Velopharyngeal insufficiency			x										1	9
Lactose intolerance		x											1	9
Prominent eye			x										1	9
Thick laryngeal wall					x								1	9
Gastroesophageal reflux							x						1	9
Syndactyly between 2nd and 3rd						x							1	9

Legend: 1 – Proband, 2 – Abdelgair et al., 2013 [10] patient 1, 3 - Pridjian et al., 1995 [32], 4 – Kalayinia et al., 2019 [8], 5 – Basaran et al., 2001 [33], 6 – Dayasiri et al. 2018 [34], 7 - Mazza et al., 2010 [7], 8 - Crowe et al., 1997 [35], 9 - Thomas et al., 2004 [36], 10 - Lessick et al., 1988 [37], 11 - Abdelgair et al., 2013 [10] patient 2

Certainly, early diagnosis can improve the patient’s quality of life through management and treatment adapted to the patient’s subjective conditions. As shown in Table 1, our patient has facial dysmorphia, developmental delay/ growth retardation, body/facial asymmetry, cardiac abnormalities, epicanthus, low set ear, spots on the skin, dysplastic/hypoplastic nails, genital abnormalities, ears with posterior rotation, body/ limb/face hypoplasia, prominent forehead, slight hypotonia, retrognathia, webbed curved neck, ptosis, scoliosis/kyphosis, down-slanting palpebral fissures, squint, hypothyroidism, myopia, insect sting allergy, asthma, bronchitis, lactose intolerance, which represents 50% of the characteristics already described for mosaic trisomy 22. Given the patient’s survival, and the quality of life associated with multidisciplinary treatment, including speech therapy, physiotherapy, psychotherapy, growth monitoring by an endocrine specialist, it can be concluded that the patient has a good prognosis. Future molecular studies could help elucidate if a genetic basis or complex trait are associated with the good prognosis observed in the described proband.

## Conclusions

This case report is the first to describe a patient with prolonged survival with the occurrence of mosaic trisomy 22 and chromosome 9 inversion. While inv(9) appears to have no phenotypic effects, it may have led to the post-meiotic event of mosaicism observed. Despite not being possible to infer the clinical characteristics associated with mosaic trisomy 22, this study highlighted many clinical characteristics already reported in other cases that are compatible with life (Table 1). This is an alert for genetic counselors and prenatal diagnosis of the need to differentiate complete to mosaic trisomy 22 and its associated survival. Finally, to reduce the underreporting risks, in case of clinical suspicion of mosaic trisomy of chromosome 22, a higher metaphase count is recommended when necessary. Herein, 10% of the cells showed 22 mosaicisms, confirming the initial hypothesis. So, we did not evaluate different tissues due to the local guidelines and family choice in not procuring further testing. Therefore, one limitation of this case study is that we cannot confirm if there’s a higher percentage of mosaicism in other tissues. Overall, independent of the number of cells evaluated, it is suggested for neonatologists to request karyotyping or other genetic testing when considering possible chromosomal genetic anomalies, especially if different dysmorphisms are observed in the newborns.

In general, the patient presented here show good prognosis, but we still lack the potential genetic or complex trait associated with the phenotype. Therefore, molecular genetic studies associated with the affected tissues are necessary to elucidate the condition. Overall, Table 1 help define potentially common phenotypic traits that can be used to guide genetic counselors with clinical profiling in databases of rare genetic conditions.

## Data Availability

All data generated or analyzed during this study are included in this published article and related files.
